# Effect of a weight loss intervention on anthropometric measures and metabolic risk factors in pre- versus postmenopausal women

**DOI:** 10.1186/1475-2891-6-31

**Published:** 2007-10-25

**Authors:** Peter Deibert, Daniel König, Mara Z Vitolins, Ulrike Landmann, Ingrid Frey, Hans-Peter Zahradnik, Aloys Berg

**Affiliations:** 1 Department of Rehabilitation, Prevention and Sports Medicine, Centre for Internal Medicine, University Hospital, Freiburg Germany; 2 Department of Obstetrics and Gynecology; Clinic for Endocrinology and Reproductive Medicine, University Hospital, Freiburg Germany; 3 Division of Public Health Sciences, Wake Forest University School of Medicine, Medical Center Blvd., Winston-Salem, USA

## Abstract

**Background:**

The present study examines changes in body weight, fat mass, metabolic and hormonal parameters in overweight and obese pre- and postmenopausal women who participated in a weight loss intervention.

**Methods:**

Seventy-two subjects were included in the analysis of this single arm study (premenopausal: 22 women, age 43.7 ± 6.4 years, BMI 31.0 ± 2.4 kg/m^2^; postmenopausal: 50 women, age 58.2 ± 5.1 years, BMI 32.9 ± 3.7 kg/m^2^). Weight reduction was achieved by the use of a meal replacement and fat-reduced diet. In addition, from week 6 to 24 participants attended a guided exercise program. Body composition was analyzed with the Bod Pod^®^. Blood pressures were taken at every visit and blood was collected at baseline and closeout of the study to evaluate lipids, insulin, cortisol and leptin levels.

**Results:**

BMI, fat mass, waist circumference, systolic blood pressure, triglycerides, glucose, leptin and cortisol were higher in the postmenopausal women at baseline.

Both groups achieved a substantial and comparable weight loss (pre- vs. postmenopausal: 6.7 ± 4.9 vs 6.7 ± 4.4 kg; n.s.). However, in contrast to premenopausal women, weight loss in postmenopausal women was exclusively due to a reduction of fat mass (-5.3 ± 5.1 vs -6.6 ± 4.1 kg; p < 0.01). In premenopausal women 21% of weight loss was attributed to a reduction in lean body mass.

Blood pressure, triglycerides, HDL-cholesterol, and glucose improved significantly only in postmenopausal women whereas total cholesterol and LDL-cholesterol were lowered significantly in both groups.

**Conclusion:**

Both groups showed comparable weight loss and in postmenopausal women weight loss was associated with a pronounced improvement in metabolic risk factors thereby reducing the prevalence of metabolic syndrome.

## Introduction

The NHANES III surveys, conducted from 1988–1994, have provided evidence that more than 50 % of women in the United States are overweight or obese [1]. Although the reasons for overweight and obesity in women are multilayered, it is well established that menopause is associated with weight gain and an unfavourable alteration in body composition [2,3]. The risk for atherothrombotic diseases in women increases after menopause. Several investigations have demonstrated that deposition of visceral adipose fat is elevated in the postmenopausal state [4]. Visceral fat accumulation has been associated with increased metabolic risk factors and hence an increase in cardiovascular diseases [5,6]. Therefore, successful strategies for reducing overweight and improving metabolic risk factors in women, particularly after menopause, are of utmost importance.

There is widespread consensus that lifestyle changes focused on improving dietary intake and increased daily physical activities, are the cornerstones in both prevention and treatment of obesity and the metabolic syndrome [7-9].

Most recently, the results of the Women's Health Initiative Trial including 48.835 postmenopausal women aged 50–79 (mean follow-up 8.1 years) were published [10]. The results show that an intensive diet modification program together with individual counselling sessions to reduce fat intake to 20 % of calories and to increase the intakes of vegetables and fruits, did not significantly reduce clinical endpoints for cardiovascular disease or stroke [11]. Weight loss was not part of the intervention and cardiovascular risk factors were rather modest. As the alterations in CVD risk factors were modest, the authors concluded that a more focused intervention of dietary and lifestyle factors may be needed to improve cardiovascular risk factors and to reduce the incidence of cardiovascular disease.

In the present study we investigated the combined effects of a weight loss regimen utilizing a meal replacement (soy-yoghurt-honey preparation) and a guided physical activity program on anthropometric measures and metabolic risk factors in pre- and postmenopausal women. In contrast to other studies published so far, we investigated whether such an intervention would produce different results in pre- versus postmenopausal women.

## Methods

Seventy-six women were enrolled in this 12 month intervention. Inclusion criteria: Female gender, age between 18 and 75 years, BMI between 27 and 35 and absence of contraindications to exercise. All subjects completed a comprehensive medical examination and routine blood tests. Subjects were excluded if they had diabetes mellitus type II, taking lipid-lowering drugs or medications that affected body weight. 22 participants were pre-menopausal, 50 were classified postmenopausal. Classification of menopause was performed by anamnesis and analysis of LH, FSH and estradiol. If FSH and LH were higher than 20 IU/ml and estradiol < 30 pmol/l, participants were classified as postmenopausal. Metabolic syndrome was diagnosed according to the NCEP ATP III criteria [12].

Written informed consent was given by all subjects; the study protocol was approved by the ethical committee of the University of Freiburg.

The study was divided into two phases; the first 6 month was designated as the active intensive intervention period where subjects had to change their dietary behaviour and increase physical activity. In the following 6 month participants were encouraged to continue these lifestyle changes however they did so without study staff supervision.

### Diet

Subjects were instructed to replace two daily meals with soy-yoghurt honey drink (Almased^® ^: amount per serving: calories 180 kcal, total fat 1.5 g, carbohydrates 16 g, protein 25 g, cholesterol 0 g) for the first 6 weeks, followed by the replacement of one daily meal for the next 18 weeks. After this period, subjects were free to use the meal replacement for the rest of the study. During the study, participants were instructed to limit fat intake to not exceed 60 g/d. Subjects were instructed during two face-to face counseling sessions and were given educational pamphlets further detailing how to improve dietary behaviour. The regimen contained about 1000 kcal/d during the first 6 weeks and 1500 kcal/d for the next 18 weeks. Participants monitored their dietary intake using 3-day food records at the beginning of the study and at 3 and 6 months.

### Physical activity

Subjects attended twice weekly 60 minute endurance physical activity sessions during the first six months. The activity sessions were supervised by a trained exercise counsellor and lasted for 6 months. Compliance was assessed by participant attendance at the sessions. Adherence was good as participants attended more than 80% of the sessions. The training program consisted of mainly aerobic or endurance type activities such as pulse-controlled walking and team sports.

Data collected at baseline and 12 months were body weight, fat mass, fat free mass, hip and waist circumference, blood pressure and blood collection for serum lipids, plasma glucose, insulin, leptin, cortisol and C-reactive protein.

### Anthropometrics

Height was measured while subjects were barefoot. Weight was measured using a digital scale, with the participants wearing light clothing or underwear, and was recorded to the nearest 0.01 kg. BMI was calculated as weight divided by height squared (kg/m^2^). Waist circumference was measured at the level midway between the lowest rib margin and the iliac crest to the nearest 0.5 cm. For body composition, the technique of the air displacement plethysmography was used (Bod Pod^®^) [13].

### Clinical measurements

Blood pressure was measured three times in the right arm in the sitting position using a standard mercury sphygmomanometer, with at least 5 minutes rest between measurements. Korotkoff's phases I and V sounds were recorded for systolic (BPsys) and diastolic (BPdia) blood pressure, respectively.

### Serum analyses

Cortisol, Insulin and Leptin were measured by commercially availably ELISA-tests (DSL Deutschland GmbH, Sinsheim, Germany). All other laboratory analyses were done in the central laboratory of the University hospital using clinical routine methods.

### Statistical methods

Normality of all variables was tested before statistical analyses using the Kolmogorov-Smirnov test procedure. Testing for changes between examination at baseline and at examination after 48 weeks was done by paired sample T-test. The two sample T-test was used to establish significant differences between the two groups at both examinations. All P values were two-sided and a P value of 0.05 or less was considered to indicate statistical significance. Analysis was conducted with the use of SPSS software (version 13.0).

## Results

A total of 72 subjects (age 53 ± 9 y; weight 86.6 ± 10.3 kg; BMI 32.3 ± 3.4 kg/m^2^,) completed the 12 month study. Participant dropout (n = 4) was not associated with side effects or adverse events. The descriptive characteristics of the subjects at baseline and 48 weeks are summarized in Table 1 and 2.

**Table 1:** Individual and Anthropometric characteristics of subjects

	**Premenopausal women (n = 22)**		**Postmenopausal women (n = 50)**	
**Variable**	**Baseline** Mean ± SD	**48 weeks** Mean ± SD	**Baseline** Mean ± SD	**48 weeks** Mean ± SD

Age (years)	43.7 ± 6.4^a^		58.2 ± 5.1^a^	
Height (cm)	166 ± 6		163 ± 6	
Weight (kg)	85.5 ± 8.6	78.8 ± 9.7^§^	87.1 ± 11.1	80.4 ± 10.7^§^
BMI (kg/m^2^)	31.0 ± 2.4^a^	28.6 ± 3.1^§^	32.9 ± 3.7^a^	30.3 ± 3.7^§^
Waist circumference (cm)	99 ± 7^a^	92 ± 9^a§^	104 ± 9	98 ± 10^a§^
Hip circumference (cm)	112 ± 6	106 ± 10^§^	116 ± 8^a^	111 ± 8^§^
Fat mass (kg)	37.4 ± 6.9^a^	32.0 ± 9.0^§^	42.5 ± 8.6^a^	35.9 ± 8.3^§^
Fat free mass (kg)	48.2 ± 4.6^b^	46.7 ± 4.5^§^	44.6 ± 5.1^b^	44.5 ± 5.2

^a ^= p < .05 between groups ^b ^= p < .01 between groups.

^§ ^= p < .01 within groups

**Table 2:** Cardiovascular risk factors of subjects

	**Premenopausal women (n = 22)**		**Postmenopausal women (n = 50)**	
**Variable**	**Baseline** **Mean± SD**	**48 weeks** **Mean ± SD**	**Baseline** **Mean ± SD**	**48 weeks** **Mean ± SD**

Total cholesterol (mg/dl)	223 ± 39	201 ± 37^b§^	234 ± 40	225 ± 36^b#^
Triglycerides (mg/dl)	115 ± 44^a^	101 ± 41^a^	163 ± 101^a^	134 ± 74^a§^
HDL-cholesterol (mg/dl)	66.4 ± 13,7^a^	69.6 ± 15.3	60.7 ± 14.9^a^	63.9 ± 13.2^#^
LDL-cholesterol (mg/dl)	124.0 ± 21.1	102.6 ± 24.8^b§^	131.2 ± 29.1	124.8 ± 27.0^b#^
Systolic BP (mmHg)	134 ± 12^a^	130 ± 15	146 ± 18^a^	135 ± 14^§^
Diastolic BP (mmHg)	86 ± 8	84 ± 7	89 ± 8	84 ± 8^§^
Glucose (mg/dl)	92 ± 14^a^	88 ± 11^a^	97 ± 12^a^	94 ± 11^a#^
Insulin (μU/ml)	9.7 ± 5.8	9.7 ± 9.8	9.6 ± 7.9	7.5 ± 7.0^#^
Cortisol (μg/dl)	17.7 ± 7.7^a^	15.4 ± 4.1^a^	22.1 ± 9.0^a^	18.0 ± 6.2^a§^
Leptin (ng/ml)	47.6 ± 15.5^a^	38.3 ± 15.9^§^	63.6 ± 27.0^a^	45.9 ± 22.4^§^

^a ^= p < .05 between groups ^b ^= p < .01 between groups.

^# ^= p < .05 within groups ^§ ^= p < .01 within groups

### Anthropometric measurements

**Figure 1:**
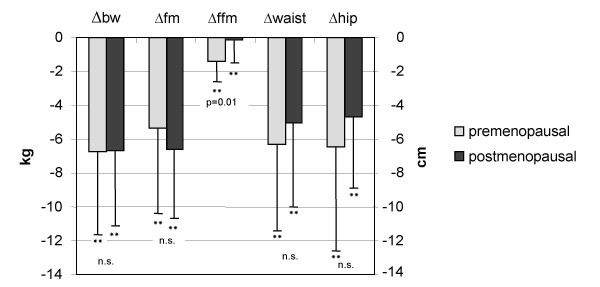
Comparison of changes after 48 weeks of body weight (Δbw), fat mass (Δfm), fat-free mass (Δffm), waist (Δwaist) and hip (Δhip) circumference between pre- and postmenopausal women. * significant changes from baseline within group p < 0.05. ** significant changes from baseline within group p < 0.01.

Weight loss was significant in both groups (premenopausal: -6.7 ± 4.9 kg, p < 0.001; postmenopausal: -6.7 ± 4.4 kg p < 0.001; Table 1) and comparable (Figure 1). At baseline, BMI, fat mass and waist circumference were significantly higher in postmenopausal women, whereas fat free mass was significantly lower compared to premenopausal women. In premenopausal women, most of the weight loss was due to a reduction of fat mass. 1.4 ± 1.2 kg of body weight reduction in premenopausal women and 0.1 ± 1.0 kg in postmenopausal women was lost due to a reduction of fat free mass (Table 1). The difference in change in lean body mass was statistically significant between the two groups (p < 0.01). Waist and hip circumferences were significantly reduced in both groups (p < 0.01 for both parameters and groups).

At baseline, systolic (BPsys) blood pressure was significantly higher in postmenopausal women (135 ± 12 to 146 ± 18 mmHg, p < 0.05), whereas diastolic blood pressure (BPdia) did not differ significantly. Improvement of BPsys only reached statistical significance (-11.3 mmHg, p < 0.001 to baseline) in postmenopausal females. Changes in BPsys and BPdia were comparable at the end of the study between the groups.

### Metabolic risk factors

**Figure 2:**
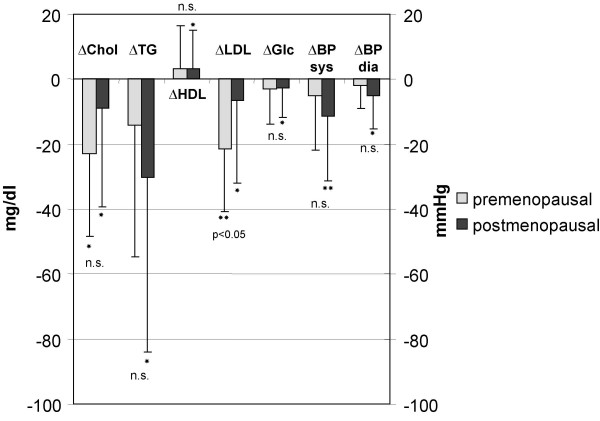
Comparison of changes after 48 weeks of total cholesterol (ΔTC), triglycerides (ΔTG), HDL-cholesterol (ΔHDL-C), LDL-cholesterol (ΔLDL-C), systolic (ΔBPsys) and diastolic (ΔBPdia) blood pressure and fasting serum glucose (ΔGluc) between pre- and postmenopausal women. * significant changes from baseline within group p < 0.05. ** significant changes from baseline within group p < 0.01.

At closeout, total cholesterol (TC) (-22.9 ± 25.5 mg/dl; p < 0.01) and LDL-cholesterol (LDL-C) (-21.5 ± 19.27 mg/dl; p < 0.01) were markedly lower in the premenopausal women but only slightly so in the postmenopausal women (TC: -8.8 ± 30,7; p < 0.05; LDL-C: -6.6 ± 25.4 mg/dl, p < 0.05). HDL-cholesterol (HDL-C) (+3.43 ± 11.7 mg/dl; p < 0.05), TG (-30.4 ± 53.7 mg/dl; p < 0.01) and fasting glucose levels (Gluc) (-2.6 ± 9.3 mg/dl; p < 0.05) improved significantly only in postmenopausal women. The reductions in TC, TG and Gluc were not significantly different between the groups (Figure 2), however the level for each parameter was still significantly lower in the premenopausal group at the end of the study (p < 0.05).

At the beginning of the study, 5 of 22 premenopausal women fulfilled the criteria of the metabolic syndrome compared to 21 of 50 postmenopausal women. After the intervention, 4 premenopausal and 8 postmenopausal women remained classified as having metabolic syndrome. The reduction of metabolic syndrome in postmenopausal women was statistically significant (p < 0.01).

### Hormones

Post intervention levels of insulin, leptin, cortisol improved significantly in the postmenopausal participants.

**Figure 3:**
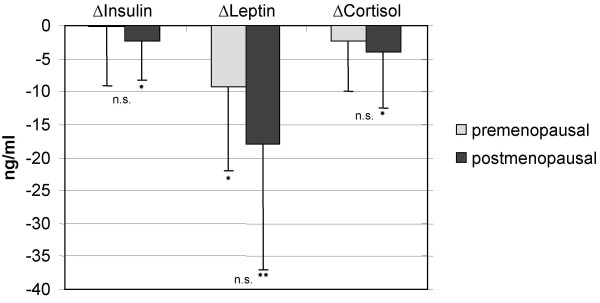
Comparison of changes after 48 weeks in Insulin (ΔInsulin), Leptin (ΔLeptin) and Cortisol (ΔCortisol) between pre- and postmenopausal women. Cortisol levels were divided by 10. *significant changes from baseline within group p < 0.05. ** significant changes from baseline within group p < 0.01.

Insulin did not change significantly during the study in premenopausal women. Cortisol was significantly higher in postmenopausal women (17.7 ± 7.7 vs 22.1 ± 9.0 ng/ml) at the beginning of the study (p < 0.02 between groups) and showed a marked 18% decrease during the study (p = 0.01) (Figure 3). In premenopausal women cortisol levels did not change significantly. Leptin was much higher in the postmenopausal group at the beginning (p < 0.02). In both groups the leptin level was significantly reduced (premenopausal: -9.3 ± 12.7 ng/ml; p < 0.01; postmenopausal: -18.1 ± 18.9 ng/ml; p < 0.01), however the difference of reduction between the two groups was not statistically significant.

Dietary intake data were collected by a self-reported 24-hour recall and a dietary record for 3 days at the beginning and after 3 and 6 months. Qualitative assessment of food records indicated that most subjects complied with the dietary program. However, dietary records revealed a considerable underreporting of the caloric intake compared to the achieved weight loss. The discrepancy was in such a way that the results of the dietary records were not considered in the analysis.

## Discussion

We found that the 12 month weight loss intervention utilizing meal replacements and dietary counselling to reduce dietary fat intake and increasing physical activity led to substantial and significant favourable reductions in anthropometric and metabolic risk factors in both pre- and postmenopausal women.

The amount of weight loss in both groups was similar to other reported findings in premenopausal [14-16] and postmenopausal [17-20] women following comprehensive lifestyle interventions. The results further demonstrate that despite comparable weight loss, the intervention was associated with a considerably higher reduction in metabolic risk factors in postmenopausal women compared to premenopausal women.

In both groups the reduction of body weight was mainly due to a reduction in fat mass. In premenopausal women loss of fat free mass accounted for 21% of the total weight reduction, whereas in postmenopausal women the loss of muscle mass was rather small (< 3% of weight loss). This is encouraging as it demonstrates that weight reduction using this type of intervention in postmenopausal women is feasible without significantly reducing lean body mass. In the context of weight loss, the preservation of muscle mass is particularly important for maintenance of motor competence and metabolic capacity of skeletal muscles. Additionally, we have shown this to be true in a previous study. In this intervention study we found that through the use of a meal replacement (supply of high quality proteins) and adequate physical activity regimen significant weight reduction may be reached without losing muscle mass. [21].

Fat mass and waist circumference were higher in postmenopausal women at the beginning of the study. This is in accordance with previous findings showing that weight gain during aging occurs predominantly in the abdominal region [22,23]. Furthermore, it is known that after menopause, waist circumference and visceral adipose tissue accumulation increase beyond the effect of ageing [24]. In our study, postmenopausal women exhibited a lower reduction of waist circumference compared to premenopausal women. This is in contrast to other studies employing regional analysis of body composition showing that abdominal fat and thigh fat were the regions most significantly affected by weight loss in postmenopausal women [25]. However, methods applied in this study do not allow a further discrimination between different fat regions.

Nevertheless, there is accumulating knowledge that fat mass as well as the relation between muscle mass and fat mass are associated with the expression of metabolic and cardiovascular risk factors [26,27]. In our study, postmenopausal women showed higher initial levels of systolic (BPsys) and diastolic blood pressure (BPdia), total cholesterol (TC), LDL-cholesterol (LDL-C), triglycerides (TG) plasma glucose (Gluc) and lower levels of HDL-Cholesterol (HDL-C). However, only differences for BPsys, TG and Gluc were significant. This underscores that the postmenopausal women investigated showed a considerable higher metabolic risk profile as presented in the NCEP ATP III criteria [28]. After participating in the intervention, the percentage of postmenopausal women with the metabolic syndrome was reduced from 42 to 16% (p < 0.01) compared to a non-significant decrease from 23 to 18% in premenopausal women. The significant reduction in the prevalence of the metabolic syndrome in postmenopausal women demonstrates that despite a relatively smaller reduction in waist circumference and fat mass, the benefit of these changes is considerably greater in postmenopausal women with respect to the reduction in metabolic risk factors.

However, our data also show that premenopausal women experienced a significant reduction in cardiovascular risk factors. Elevated fasting levels of plasma insulin and impaired glucose tolerance have been widely recognized as major independent risk factors for the development of coronary heart disease [29]. In both groups, fasting blood glucose was reduced. However, the reduction in insulin levels was only significant in the postmenopausal women. As insulin levels or insulin sensitivity have shown to be correlated to abdominal fat mass [30], the decrease in insulin in postmenopausal women may be explained by the higher reduction of fat mass in this group.

The same differences were detected for cortisol, one of the counterparts of insulin action. Our results confirm the finding that higher levels of adiposity are associated with higher concentrations of serum leptin [31]. Leptin levels were significantly reduced by reduction of fat mass in both groups. Despite a higher absolute reduction of fat mass in postmenopausal women, the proportion of fat mass remained higher in postmenopausal women at the end of the study. Therefore, postinterventional Leptin levels were still higher in postmenopausal women.

Doucet et al. described that during weight reduction the decrease in fasting plasma insulin was the only significant correlate of changes in fasting plasma leptin levels [32]. In our study, reduction of insulin, leptin and cortisol was severalfold higher in the postmenopausal group.

Weight loss programs including pharmaceutical interventions have shown to be associated with considerable higher drop-out rates [33]. In our study, the weight loss intervention was very well accepted by all participants as shown by the low drop-out rate. In an evaluation participants appreciated the personal counselling and the guided activity program as very helpful.

## Conclusion

The results demonstrate that the intervention, consisting of meal replacement beverage, additional dietary counselling and guided physical activity sessions, was associated with considerable weight loss in both premenopausal and postmenopausal women. In addition, after the intervention, metabolic risk factors in women were decisively lower; indicating the usefulness of this lifestyle approach to reduce both metabolic syndrome and risk factors for cardiovascular disease in women.

## Abbreviations

ATP Adult treatment panel

BMI body mass index

BPsys systolic blood pressure

BPdia diastolic blood pressure

Gluc blood glucose

HDL-C high density lipoprotein cholesterol

LDL-C low density lipoprotein cholesterol

NHANES National Health and Nutrition Examination Survey

TC total cholesterol

TG triglycerides

## Competing interests

Aloys Berg has received grants from Almased Wellness Corporation. The other authors declare that they have no competing interests.

## Authors' contributions

Aloys Berg was the principal investigator of the study. Peter Deibert and Daniel König were involved in the design and execution of the study and performed the statistical analysis. Ulrike Landmann and Ingrid Frey performed the diet counselling and physical training of the participants. Mara Z Vitolins and Hans-Peter Zahradnik contributed to data interpretation and manuscript preparation. All authors read and approved the final manuscript.
